# Canadian Notes

**Published:** 1910-07-09

**Authors:** 


### CANADIAN NOTES.

### EPIDEMIC OF TYPHOID FEVER AT THE SILVER MINES IN NORTHERN ONTARIO.

Two or three years ago silver was discovered in large quantities in a district known as Cobalt, situated in Ontario, about 300 miles north of Toronto. There was a rush to the locality, a town was built on the borders of Lake Cobalt, and at the present time there are more than 30 mines in full working order. The mining camps are outside the town, and the sanitary conditions of both the camps and towns are as bad as they possibly can be. Typhoid fever has existed in the town and camps for some considerable time, and in August last developed into a somewhat serious epidemic. According to the laws of Ontario, when a township has more than 5,000 inhabitants the State Board of Health has no authority, but it is controlled by local health officers. However, so grave did the situation become that early in September the local officials of Cobalt applied to the Provincial Board of Health of Ontario for assistance, and Dr. Chas. Hodgetts (its secretary) and Dr. Bell (one of its inspectors) were sent to Cobalt to advise and suggest remedies. They found the conditions as regards sanitation elementary and primitive in the extreme. In fact, the ordinary laws of health were wholly neglected. The water-supply was obtained from surface wells, most of which were very shallow, and proper water-closet arrangements were totally lacking. The consequence was that the wells were easily contaminated. The representatives of the Provincial Board of Health gave directions for the closing of more than 70 wells, and to the best of their ability insisted that the town and camps should be cleaned and disinfected thoroughly. They recommended to the mineowners that a pure water-supply should be provided in the mines, and that the earth-bucket system should be installed for the convenience and safety of the miners. The minowners provide hospitals and nurses for their workers, and at the present time there are in these hospitals 56 patients. In the town itself there are 41 persons suffering from typhoid. Fortunately the disease has been of a comparatively mild type, and the death-rate has not been especially high. Nevertheless, there are no signs of abatement; the number of cases in the town since August has exhibited a steady increase, while the number in the camps has shown no decrease. It is interesting to note that during August and September flies were present in immense quantities in the Cobalt district.

